# Does Economic Overheating Provide Positive Feedback on Population Health? Evidence From BRICS and ASEAN Countries

**DOI:** 10.3389/fpubh.2021.661279

**Published:** 2021-03-18

**Authors:** Chi-Wei Su, Shi-Wen Huang, Ran Tao, Muhammad Haris

**Affiliations:** ^1^School of Economics, Qingdao University, Qingdao, China; ^2^Qingdao Municipal Center for Disease Control and Preventation, Qingdao, China; ^3^Department of Business Administration, National Fertilizer Corporation Institute of Engineering and Technology, Multan, Pakistan; ^4^Institute of Banking and Finance, Bahauddin Zakariya University, Multan, Pakistan

**Keywords:** business cycle, population health, BRICS, ASEAN, panel unit root, panel threshold regression model

## Abstract

This paper explores the relationship of real GDP per capita with cancer incidence applying panel threshold regression model in BRICS and ASEAN countries. The empirical results highlight that the business cycle has an inverted-U correlation with population health indicators and a non-linear single threshold effect. In BRICS countries, the health-promoting effect of economic growth is significantly weaker when exceeding the threshold. Similarly, economic growth in ASEAN countries, even worsens population health, after the turning point. These asymmetric effects are strongly related to the response of regional economic globalization health policies. Changes in economic expansion and overheating may have serious adverse effects on health care systems in emerging economies. Governments should adopt more aggressive health care policies during economic overheating, to avoid wasting health care resources.

## Introduction

The main purpose of this paper is to explore whether the population health (describes as cancer incidence, CI) in emerging economies respond to business cycles (describes as real GDP per capita, GDP), and how does economic growth or economic overheating affect population health. With the continuous accumulation of economic growth on the medical industry capital, the positive correlation between economic growth and population health appears more intuitively in the process of economic development (1, 2). BRICS and ASEAN countries,^1^ as representatives of emerging economies, have made profound changes in their health care systems to adapt to the huge demographic, political, economic and socio-cultural transformations brought about by their economic growth (3). As a result, business cycles may affect population health in emerging economies countries (4), as evidenced by the health care transformation triggered by investments in the health care industry, which is reflected in the declining cancer incidence in past 20 years. The economic growth may lead to lower mortality (or in better health) can be explained in two ways. First, an increased GDP allows patients to invest more in their own health (5, 6). Second, continued economic growth has increased the professionalism of medical equipment and services in emerging economies, which is also reflected in social welfare (7). In turn, improvements in health have direct positive feedback on economic growth through higher productivity and more hours worked (8). Therefore, the complex behavior of economic growth may have a substantial impact on resident health in emerging economies (9, 10). This study can benefit the decision-makers to focus on the harmful effects of excessive economic growth on the health care system. The government should intervene more to promote the stable development of the medical industry when the economy is overheating.

Since the work of Pritchett and Summers (11), existing studies have discussed whether economic growth improves health and vice versa. The mainstream view Brenner (12–14) holds that there is a mutually promoting relationship between these two variables. Ruhm (15–17) finds a procyclical variation in mortality with the business cycle. Alleyne and Cohen (18) and Lorentzen et al. (19) also demonstrate this. However, some contrary evidence suggests that economic recessions can also reduce mortality. Neumayer (20), Tapia Granados (21), Gerdtham and Ruhm (22), Lin (23) show that growth in GDP also stimulates cancer rates in some countries. Although this series of studies reflect asymmetries in the impact of economic growth or recession on health indicators, most of these findings have occurred in high-income countries (24). Emerging economies have shaped the world landscape in the past two decades that the impact of the business cycle on population health is unclear. In recent years, Liu and Huang (3) propose an asymmetrical relationship between short-term life expectancy and economic growth in ASEAN sample. Although previous studies have included several developing countries, the question of whether overheating will inhibit national health has not been well-addressed. In addition, the periodicity of population health changes with the inclusion of more recent data (25, 26). Especially with the recent outbreak of coronavirus disease 2019 (COVID-19), breakpoints, as a key feature, can lead to biases and pseudo-regressions (27, 28). The emerging economies selected for this paper have experienced rapid economic recession and rise, and the combination of panel threshold regression and panel unit root test attribution increases the reliability of the findings in describing the dependent variable, outliers and non-linear distribution (29). The question of whether economic overheating inhibits population health is effectively answered.

Emerging countries have undergone tremendous demographic, political, economic, and sociocultural transitions along with rapid economic development (30). Among them, the total GDP in ASEAN has grown more than fivefold from 1999 and becoming the world's fifth-largest economy (31). The total GDP of the BRICS countries has exceeded even 2.1 billion dollars since 2019. Economic development in the BRICS and ASEAN region has gone through several phases of rapid economic fluctuation, which facilitates the assessment of the impact of different economic expansions and recessions. Therefore, this paper focuses on two representative international economic organizations in emerging economies, BRICS and ASEAN countries, to compare and analyze the characteristics of their population health impact factors. The transformation of modern advanced economies also presents many challenges to the health care system, and governments should mitigate the adverse effects of economic fluctuations on population health. Abnormal fluctuations in economic development often affect the uncertainty of countries' health care policies, especially during crises (32). According to the World Health Organization in 2018, the rate of healthcare spending in BRICS countries is 8%, with the fastest increase in the share of healthcare spending in emerging economies. However, high health care expenditure growth was accompanied by a decline in premature non-communicable disease (cardiovascular disease, cancer, diabetes, chronic respiratory diseases) mortality, from −1.6% in 2000–2010 to −1.1% in 2010–2016. This has caused researchers to worry about the negative effects of an overheated economy on population health. In addition, the entry of large amounts of state capital into the health care market has had a profound impact on improving the national health care system. The complex volatility of the business cycle has an impact on the population's healthcare investment and importance through income and substitution effects (33, 34). In addition, because emerging market healthcare systems are immature, they are subject to greater pressure from rapidly changing business cycles (35, 36). To address the shortcoming that associations between variables may be affected by external factors and exhibit non-linear characteristics (37), this paper explores whether population health is influenced by economic growth applying a panel threshold regression model. Also, we further investigate whether this linkage is asymmetric and find new evidence on factors affecting health, filling in the gaps in these issues.

While the positive correlation between the two variables is most intuitively evident in the economic development process, the role of the business cycle on population health (mortality) remains ambiguous and controversial in emerging economies. And does sustained economic growth (even overheating) also improve health? Will there be a threshold effect in the panel sample from BRICS and ASEAN countries? Therefore, this paper makes the following contributions to the study of the above issues. Firstly, the empirical results find that BRICS and ASEAN countries have an inverted U-shaped relationship on the defined threshold, which may be caused by the decline of marginal utility of economic growth and the dominance of substitution utility. This threshold effect means that economic overheating does not bring the same improvements to health care but rather wastes it. Secondly, by comparing the same relationship with high-income countries, we find that the relevant of consumer price index on health different from the health utility model (15) of which heterogeneity is only for ASEAN. This implies that inflation in ASEAN countries has increased the burden of health care on individuals. And the stronger role of government intervention in BRICS countries is well-implemented in the establishment of health care delivery systems. Thirdly, because uncertainty in external factors may affect the relationship of variables (37, 38), this paper considers a panel threshold regression model to explore the non-linear characteristics of population health and economic growth. Also, we further investigate whether this linkage is asymmetric. These supplement the defects of the linear hypothesis in the previous literature.

The rest of the paper is structured as follows. Section “Literature Review” reviews the existing literature, whereas Section “The Health Utility Model With Economic Growth” presents the health model. Section “Methodology” describes the econometric approach employed in this paper. Section “Data” is the data description. Section “Empirical Results” shows the findings of the study. Section “Conclusions” offers concluding remarks.

## Literature Review

In the process of economic development, the positive correlation between the business cycle and resident health is observed. Brenner (12) indicates that economic fluctuation has a negative impact on the mortality rate of heart disease. Chen (1) and Tapia Granados and Ionides (25) find a biologically directed causal relationship between the business cycle and population health. Biggs et al. (39) note that public health changes synchronously with the economic cycle, but this procyclical effect is affected by individual income. Friedman (40) and Svensson and Krüger (41) also propose that 30% of progress in medical treatment can be explained by specific economic instability, and business cycles have long-term effects on population health. Swift (42) argues that the capital redistribution caused by economic development is an important factor affecting the health-care system. He et al. (43) give the evidence of the non-linear effect business cycles and population health in China. Liu and Huang (3) evidence that there is an asymmetric influence on business cycles and population health.

However, this view is not always confirmed, Ruhm (15–17, 44) studies fixed utility models based on panel data and find that mortality is procyclical as the business cycle moves. Dollar and Kraay (45) argue that income inequality caused by economic growth will worsen the healthy productivity of the poor. Svensson (46) believe that the rise of GDP in Sweden has a temporary effect on health and hard to observe. McInerney and Mellor (47) estimate that the converse periodicity of youth mortality was contrary to the results of the elderly and children samples.

Intuitively, the promotion effect of the improvement of the health level on economic development is also rational and reliable. Davlasheridze et al. (8) argue that healthier levels contribute to increased work efficiency and productivity. Bloom and Canning (48) propose that the improvement of health status and economic development tend to promote each other. Weil (49) observes that health can serve as a basis for large disparities between rich and poor countries. Although the literature has focused more on the positive macroeconomic effects in health levels, there are also authors like Tapia Granados (21) that indicate that general health improvement conditions are not so crucial in determining economic growth. Acemoglu and Johnson (50) further point out that the influence of GDP on population health could be negligible in the long run.

The relationship of the theory of bidirectional causality between the business cycle and population health is also supported by the mainstream view (9, 10). Weil (51) suggests that healthier individuals are likely to work more hours and be more productive and that higher national income may simultaneously lead to improvements in personal nutrition and public health infrastructure. Liu and Huang (3) further provide evidence of the non-linear and asymmetric relationship of COP on EPU. Bloom and Canning (48) note that there is a continuing two-way causality between business cycles and national health systems. Chen (1) supports the hypothesis of a two-way causal relationship between GDP and population health in the equilibrium state, and it manifests the information gains between markets. Barro (52) constructs an extended theoretical framework of the neoclassical growth model to illustrate the two-way causality between health status and economic growth. Lam and Piraerard (53) demonstrate that the correlation between the health status of the U.S. population and the business cycle varies over time. In summary, studies conducted in different countries and over different periods have yielded mixed results. In addition, it is unclear whether there is a threshold effect between economic development and population health. Are economic overheating and health still procyclical? This paper will make an in-depth study of the above issues.

## The Health Utility Model With Economic Growth

We refer to Ruhm's model (15) to describe the effects of economic growth on population health. The maximize of the utility function is set to *U*(*H, Z*), where *H* is health, *Z* is general consumer products. *H* depends on medical care (*M*), non-work time (*R*), and baseline status (*B*), with *H*_B_, *H*_R_, and *H*_M_ >0 (The subscript is the partial derivative). The budget is *Y* = *WL* = *P*_Z_*Z* + *P*_M_*M* and time constraints are for *R* = *T*−*L* (*Y, T, L, W, P*_Z_, and *P*_M_ indicating income, available hours, work hours, the hourly wage rate, and consumption good price and medical care price, respectively).

Given L, the optimization problem can be expressed by the following:

(1) $$\begin{array}{l} max_{M,R,Z}\;=\;U\left(H\left(M,R,B\right),Z\right)\text{ subject to }W\left(T-R\right)\\ {\text{ = P}}_{Z}{\text{ + P}}_{M}\text{M} \end{array}$$

Such a first-order condition implies that we need to find *M, R*, and *Z* such that *U*_H_*H*_M_/*P*_M_ = *U*_H_*H*_R_/*W* = *U*_Z_/*P*_Z_ holds.

Economic growth leads to possible changes in the relative price of health care services, and baseline health may rise or fall. In addition, wage rates may rise. a decrease in *P*_M_ will increase optimal health. If substitution effects dominate, higher wages will increase the desired hours of work. This worsens health by increasing the time cost of maintaining health activities but also increases earnings. Thus, the overall effect of economic growth on health is unclear.

If *Z* has a direct effect on health, the utility function becomes *H* = *H* (*M, R, B, Z*) and the optimization problem is *U*_H_*H*_M_/*P*_M_ = *U*_H_*H*_R_/*W* = (*U*_H_/*H*_Z_ + *U*_Z_)/*P*_Z_. *Z* has an indirect effect on utility. W generally changes in the same direction as *M* and *Z*. If *H*_Z_ > 0, *H* is more likely to decline compared to when *H*_Z_ = 0. Conversely, a decrease in income is more likely to improve health if *H* < 0.

Following the demand for health theory, we set the baseline state at time *t* to be :

(2) $$\begin{array}{r} B_{t}=B\left(B_{t-1},M_{t-1},\delta ,\varepsilon_{t}\right)=\left(1-\delta \right)B_{t-1}+m_{t-1}+\varepsilon_{t} \end{array}$$

where δ is the depreciation rate of health capital, ε is stochastic shock, and *m* = *M*^α^, with 0 < α <1. After substitution into the equation, we get:

(3) $$\begin{array}{r} B_{t}={\left(1-\delta \right)}^{n}B_{t-n}+\sum_{i=0}^{n-1}{\left(1-\delta \right)}^{i}\left(m_{t-i-1}+\varepsilon_{t-i}\right) \end{array}$$

If *n* is large, ${\left(1-\delta \right)}^{n}B_{t-n}$ approaches zero and the baseline state at *t* is influenced by health care investment and shocks. Changes in income and time costs affect the current and future health care delivery.

## Methodology

### Tests of Gibrat's Law

We construct a basic model to test the influencing factor of healthy and verify whether the economic growth rate of emerging countries conforms to Gibrat's law (54). In conjunction with Yu et al. (55), the original Gibrat's law is formally formulated as follows:

(4) $$\begin{array}{r} \frac{S_{i,t}-S_{i,t-1}}{S_{i,t-1}}=\varepsilon_{i,t} \end{array}$$

where *S*_*t*_ is the economic growth rate during the period of *t*. ε_*t*_ follows a normal distribution. Gibrat's law states that the variation in the explanatory variable is independent of the size of the independent variable. Therefore, if the law holds, the health level will have a log-normal distribution. This means that the proportional probability of change in growth over time should be the same for all countries within an economic organization in a given period. However, this has no relation to its economic development in the initial period. Gibrat's law is verified by the following verification formula for random walk compliance:

(5) $$\begin{array}{r} \Delta \log\left(S_{i,t}\right)={\alpha_{i}+\beta_{i}\log\left(S_{i,t-1}\right)+\varepsilon }_{i,t} \end{array}$$

where the null hypothesis is: β_*i*_ ≤ 0. To calculate β_*i*_, we utilize two panel unit root tests.

Previous empirical studies have explored the applicability of Gibrat's law, but conflicting views remain. Some early studies did not support a positive relationship between enterprise economic growth and population health (16, 17). Other studies further find that a positive or negative relationship between the two variables does not necessarily affect the applicability of Gibrat's law (55, 56). Recent studies have proposed that the validity of Gibrat's law varies with different sample sizes (57), years of observation (58). Based on previous studies, we consider Gibrat's law to investigate the economic growth rate of emerging countries.

### Panel Threshold Regression Model (PTRM)

We refer to the panel single threshold regression model of Hansen (59). {*c*_*it*_, *GDP*_*it*_, *x*_*it*_:1 ≤ *i* ≤ *n*, 1 ≤ *t* ≤ *T*}, by establishing the following single threshold model:

(6) $$c_{it}=\{\begin{array}{c} \mu_{it}+\beta_{1}GDP_{it}+\alpha_{1^{\prime }}x_{it}+\varepsilon_{it},\;if\;GDP_{it}\leq \gamma \\ \mu_{it}+\beta_{2}GDP_{it}+\alpha_{2^{\prime }}x_{it}+\varepsilon_{it},\;if\;GDP_{it}>\gamma \end{array}$$

where *GDP*_*it*_ is the growth rate of real *GDP* per capita as the threshold variable; *c*_*it*_ is the cancer incidence; γ is the estimated threshold value; β_1_ and β_2_ are the threshold coefficients; *x*_*it*_ is the control variable; α_1_ and α_2_ are coefficients of the control variables; and μ_*it*_ denotes the fixed effect in different countries under varying conditions. ε_*it*_ is a white noise process compliance with $\varepsilon_{it}\sim \left(0,\sigma^{2}\right)$; *i* and *t* denote the countries and time.

Equation (6) can also transform into the following formula:

(7) $$\begin{array}{r} {c_{it}=\mu }_{it}+\beta_{1}GDP_{it}\psi \left(GDP_{it}\leq \gamma \right)+\beta_{2}GDP_{it}\psi \left(GDP_{it}>\gamma \right)\\ +\alpha^{\prime }x_{it}+\varepsilon_{it} \end{array}$$

However, there may in fact be more turning points in applications. Hence, the multiple shapes of the threshold model can be expressed as Eq. (8):

(8) $$c_{it}=\{\begin{array}{c} \mu_{it}+\beta_{1}GDP_{it}+\alpha_{1^{\prime }}x_{it}+\varepsilon_{it},\;if\;GDP_{it}\leq \gamma_{1}\\ \mu_{it}+\beta_{2}GDP_{it}+\alpha_{2^{\prime }}x_{it}+\varepsilon_{it},\;if\;\gamma_{1}<GDP_{it}\leq \gamma_{2}\\ \mu_{it}+\beta_{3}GDP_{it}+\alpha_{3^{\prime }}x_{it}+\varepsilon_{it},\;if\;GDP_{it}>\gamma_{2} \end{array}$$

The regression shape of above can also show as:

(9) $$\begin{array}{l} {c_{it}=\mu }_{it}+\beta_{1}GDP_{it}\psi \left(GDP_{it}\leq \gamma_{1}\right)\\ \;+\beta_{2}GDP_{it}\psi \left({\gamma_{1}<GDP}_{it}\leq \gamma_{2}\right)\\ \;+\beta_{1}GDP_{it}\psi \left(GDP_{it}>\gamma_{2}\right)+\alpha^{\prime }x_{it}+\varepsilon_{it} \end{array}$$

where γ_1_ and γ_2_ are threshold values (γ_1_ < γ_2_). Accordingly, multiple threshold regression models can also be deduced.

Previous studies of variable relationships in the literature have been based on the assumption of linearity, and such conclusions are not convincing. Yeh et al. (37) point out that the association between variables may be influenced by external factors and exhibit non-linear characteristics. The use of PTRM obtained a non-linear and asymmetric relationship between economic growth and population health. Furthermore, the method effectively eliminates individual fixed effects, and the use of two-stage least squares confirm the results (60). However, due to the limited sample size, the use of bootstrap method to draw the sample has strict requirements on the number of replications. We choose 500 as the parameter to minimize the effect of the resulting inaccuracy on the results (61). In addition, there may not be just a single threshold effect in the actual empirical results. The existence of multiple threshold effects increases the difficulty of analyzing the relationship between variables.

## Data

The sample used the annual data from 1990 to 2019 including a total of 450 annual entries. Since cancer data for most countries were collected from 1990, the sample covers a period starting in 1990. The data sources are the National Bureau of Statistics and the Gapminder Database. Most previous studies believe that the composite cancer incidence (CI) is one of the factors that significantly affect the health of the population because the composite cancer incidence can reflect the overall medical level of a country (62, 63). Pan et al. (64) and Lim et al. (65) chose the incidence of cancer as a measure of health. In this paper, real GDP per capita (GDP) is used to measure the growth of the business cycle in each country and as a threshold variable (66–68). In general, an increase in real GDP per capita greatly increases consumption levels and expands government spending, and a large number of empirical studies have demonstrated that real GDP per capita has a positive effect on population health (5–7). However, government investment in healthcare is also an expensive activity that does not guarantee potential returns (44), which motivates to study the threshold effect of GDP. In general, business cycle adjustment has a lagged effect on population health, so the explanatory variable is selected as the prior period real GDP per capita.

Three control variables are introduced in this study. The first is the consumer price index (*CPI*), which captures changes in the price levels of consumer goods and services generally purchased by households (69, 70). Its rate of change reflects the degree of inflation or deflation (71). Individuals will weigh the costs and benefits of spending on health care to influence their health status (72). The second is the disposable income per capita (*IC*), which is considered the most important determinant of consumer spending and thus is often used to measure changes in a country's standard of living (73, 74). Ettner (75) further states that residents with higher incomes have a stable and sufficient cash flow, allowing individuals to spend more of their spending on healthcare. Finally, the service percentage of GDP (*SP*) has long been used as an indicator for developed countries (76, 77). However, the higher share of services is not better, and a rising share may or may not be the result of the industrial division of labor, rising costs, and underdeveloped manufacturing. The diversified structure of the service sector can also have an impact on the healthcare industry (78).

Table 1 divides the 15 emerging economies into BRICS and ASEAN countries for the statistical description of variables. As can be seen from Table 1, ASEAN has a higher mean composite CI rate of 18.499 per 1,000 people. This may be related to the late start of cancer treatment in some ASEAN countries. The real GDP per capita and the disposable income per capita of BRICS countries are significantly higher than those of ASEAN countries, with an average of 9,264.723 and 19,097.200, respectively. The Std. Dev. in CI variable is significantly larger in ASEAN countries, which may be due to the greater differences in economic development within the same organization. In terms of the proportion of services in GDP, the industrial structure of ASEAN countries is better, with an average of 49.5% and BRICS for 44.7%. We can also observe that the kurtosis of SP is >3 for both economic organizations, showing a thick tail. Consumer prices in both economies have risen sharply recently, and the data structure is skewed to the right. The Jarque-Bera test results indicate that all of the data series follows a normal distribution.

**Table 1:** Descriptive statistics of the variables.

		**Mean**	**Maximum**	**Minimum**	**Std. Dev**.	**Skewness**	**Kurtosis**	**Jarque-Bera**
BRICS	*CI*	18.499	25.560	12.470	4.091	0.193	1.756	1.838
	*CPI*	72.925	119.990	37.205	26.778	0.364	1.817	2.089
	*GDP*	9,264.723	11,567.000	7,154.200	1,372.790	0.225	1.794	1.796
	*IC*	19,097.200	23,597.000	15,025.300	2,668.759	0.240	1.795	1.823
	*SP*	0.447	0.481	0.421	0.013	0.398	3.942	1.648
ASEAN	*CI*	19.990	29.160	11.620	5.871	0.099	1.573	2.250
	*CPI*	68.479	136.800	17.748	34.551	0.309	2.129	1.237
	*GDP*	5,691.715	7,784.000	4,361.800	1,253.775	0.490	1.657	2.992
	*IC*	10,154.080	14,256.000	7,544.000	2,457.967	0.487	1.672	2.939
	*SP*	0.495	0.557	0.395	0.040	−0.880	3.114	3.370

## Empirical Results

We perform a panel unit root test to investigate whether population health in emerging economies is consistent with Gibrat law. Referring to Shiller and Perron (79), the one-equation ADF test performs poorly in small samples. In this paper, the tests of Levin et al. [(80), LLC] and Im et al. [(81), IPS] tests are considered to address the limited power problem. As it stands out in Table 2, there is no unit root of cancer incidence, which does not comply with Gibrat's law. Furthermore, we should conform stationary of all variables before using PTRM to avert pseudo-regression. The panel unit root tests for both LLC and IPS manifest that variables are all significant at the level of 10%. Therefore, we proceed to analyze the PTRM.

**Table 2:** Panel unit root tests.

	**Variables**	**Panel augmented Dickey–Fuller test**			
		**Levin-Lin-Chu**		**Im-Pesaran-Shin**	
		***t*-Statistic**	***p*-Value**	***t*-Statistic**	***p*-Value**
BRICS	*CI*	−4.438	0.077	−2.154	0.061
	*CPI*	−5.550	0.010	−2.541	0.007
	*GDP*	−5.241	0.091	−1.896	0.078
	*IC*	−5.060	0.071	−1.955	0.074
	*SP*	−10.758	0.000	−4.986	0.000
ASEAN	*CI*	−5.148	0.100	−1.866	0.085
	*CPI*	−8.614	0.000	−2.655	0.000
	*GDP*	−15.370	0.000	−4.809	0.000
	*IC*	−15.219	0.000	−4.635	0.000
	*SP*	−13.299	0.000	−3.663	0.000

The results recorded, highlighted in Table 3, presents an optimal level of capital structure for both BRICS and ASEAN countries. The single threshold of GDP growth rate for BRICS countries is 0.0649, which is significant at 10% level of significance and *F*-statistic is 9.2232. Also, according to Table 4, there is a significant negative correlation between GDP and CI when the economic growth rate of BRICS countries is <0.0649 and the estimated coefficient is −0.3337. The negative correlation exhibited becomes weaker when the GDP growth rate is greater than a limited threshold. It indicates that there is a single threshold effect, which makes the model show an asymmetrical non-linear relationship with inflection points.

**Table 3:** Tests for threshold effects between GDP and Health indexes.

	**Single threshold effect test**			**Double threshold effect test**			
	**Threshold value**	***F*-statistics**	***p*-Value**	**Threshold value**		***F*-statistics**	***p*-Value**
BRICS	0.0649^*^	9.2232	0.0810	0.0649	0.0752	8.8047	0.1500
ASEAN	0.0357^**^	13.2673	0.0434	0.0357	0.0438	7.2657	0.1900

** , and

* * respectively indicates significance at the 5, and 10% level*.

**Table 4:** Estimated coefficients of real GDP per capita growth in different regions.

**Region**	**Coefficient**	**Estimated value**	**OLS se**	***t_***OLS***_***	**White se**	***t_***White***_***
BRICS	${\hat{\beta }}_{1}$	−0.3337	0.0858	−3.8892***	0.0765	−4.3620***
	${\hat{\beta }}_{2}$	−0.2097	0.0889	−2.3588**	0.0694	−3.0216***
ASEAN	${\hat{\beta }}_{1}$	−0.0833	0.0372	−2.2392**	0.0178	−4.6797***
	${\hat{\beta }}_{2}$	0.1039	0.0721	1.4410*	0.0625	1.6624*

OLS se (White se) refers to homogeneous (heterogeneous) standard deviations. ***, **, and *, respectively, indicates significance at the 1, 5, and 10% level.

The health care budget of BRICS countries has increased approximately six times since 1999. In 2017, at the BRICS meeting on “Building an Integrated Health Service System for the Future,” China clarified that the share of personal health expenditure in total health costs has been reduced to <30%. The government's public spending on health care has been effective. In India and Brazil, the share of health in GDP exceeded 6 and 8% after 2015, and their public health spending exceeded 20% of total health costs, plaguing the incidence of lung and oral cancer by 2% in 10 years (82). South Africa has also developed public-private partnership mechanisms in recent years, effectively alleviating the shortage of health resources (83). Russia's health insurance foundation has made more than 1,000 commonly used drugs free of charge, significantly meeting the population's basic medical needs (84). Although economic growth can bring about improvements in public health facilities through government spending on health care, Arrive and Feng (85) still point out that health care resources are wasted in BRICS countries. According to the Endpoints News Global Drug R&D Spending 2016 ranking, Chinese drug companies spent a total of only 3.5 billion dollars on R&D. Such inefficient capital investment significantly slows healthcare outcomes. In addition, research by Sahu and Gahlot (86) suggests that the negative health correlation in BRICS countries is largely related to corruption in their healthcare systems.

In contrast, in ASEAN countries, the effect of economic growth on health is in an inverted U-shape, i.e., an overheated economy worsens health. In Table 3 when the economic growth rate exceeds the threshold value of 0.0357, the estimated coefficient is 0.1039, i.e., overheated economic growth instead increases the cancer incidence, which was not observed in previous studies.

The effect of economic growth on the reduction of cancer incidence is very obvious when the economic growth rate is less than the inflection point, which is closely related to the health expenditure of ASEAN countries (87). As of the official website of the World Health Organization in 2017, the overall share of health expenditure in ASEAN countries exceeded 4% of GDP, with Cambodia's health expenditure ratio reaching 5.9%. In addition, the 2018 ASEAN Health Cooperation Forum was held to strengthen cooperation on health emergencies and food safety and nutritional health, which effectively contributed to the long-term population health of ASEAN countries (88). But the existence of inflection points reveals problems in the health care systems of ASEAN countries (22, 23). Ruhm (44) argues that more income does not drive healthy consumption when per capita income levels increase, which is particularly evident in low-income countries. Also, according to the law of diminishing marginal health utility, excessive economic growth may lead to a greater substitution effect on health care investment than the income effect. Kimman et al. (89) show that the burden of cancer in ASEAN countries does not decrease as a result of increased income. Haseeb et al. (90) note that R&D spending on health care in ASEAN countries has been slow to improve population health and that technical barriers to health care have led to significant wastage of government funds.

For the threshold of BRICS and ASEAN countries, we can further determine the impact of each control variable on population health which we highlight (Table 5). As pointed out in Table 5, CPI has a positive effect on CI in both BRICS and ASEAN countries. Research from Dunn et al. (91) show that a rise in the CPI means a decrease in the purchasing power of goods, and likewise a decrease in spending on health care. Inflation can likewise shrink your investment in health care, which can make a big loss in residents' health insurance (92). This is particularly evidenced by the CPI of the BRICS countries. Using 2010 as a benchmark, inflation in the BRICS countries has increased at a rate >5% per year over past decade. In contrast, there is a negative correlation between SP and CI in both BRICS and ASEAN. Increasing tertiary output in emerging economies has accelerated the development of medical services, with the average SP in ASEAN countries exceeding 49.5%. Patel (93) argues that advances in medical services have increased timely patient access and cure rates, which is attributed to the large investment in medical services capital. Finally, higher IC also improves health, and people may spend more of their income on giving themselves health insurance and disease treatment (5, 6, 39). Moscone and Tosetti (94) find that higher disposable income makes the average person more concerned about his or her health status, which is reflected in the average annual health care expenditure of individuals. From a physiological perspective, higher IC leads to higher nutritional food intake, which would significantly improve health, especially in poorer areas (95, 96).

**Table 5:** Estimated coefficients of the control variables.

**Region**	**Coefficient**	**Estimated value**	**OLS se**	***t_***OLS***_***	**White se**	***t_***White***_***
BRICS	${\hat{\alpha }}_{1}$	0.0288	0.0152	1.8947*	0.0138	2.0869**
	${\hat{\alpha }}_{2}$	−0.0028	0.0015	−1.8666*	0.0017	−1.6470*
	${\hat{\alpha }}_{3}$	−0.0240	0.0203	−1.1822	0.0121	−1.9834*
ASEAN	${\hat{\alpha }}_{1}$	0.0428	0.0260	1.6461*	0.0213	2.0093**
	${\hat{\alpha }}_{2}$	−0.0106	0.0038	−2.7894***	0.0082	−1.2926
	${\hat{\alpha }}_{3}$	−0.0125	0.0054	−2.3148***	0.0079	−1.5822*

OLS se (White se) refers to homogeneous (heterogeneous) standard deviations. The estimated coefficients of ${\hat{\alpha }}_{1}$ is for consumer price index, ${\hat{\alpha }}_{2}$ is for disposable income per capita, ${\hat{\alpha }}_{3}$ is for services percent of GDP. ***, **, and *, respectively, indicate significance at the 1, 5, and 10% level.

In order to obtain more reliable conclusions, we select two new control variables to consider it in a robustness test. The new control variable includes government expenditure on health care (*GEH*), which refers to the financial allocation of governments for health undertakings (97). GEH includes funds for public health services and public medical services and often uses as a direct economic indicator of population health (98, 99). Another control variable is the investment percentage of GDP (*IP*), which is an effective measure of the size of the country's real economy (100–102). To address the endogeneity problem, we consider that the business cycle affects population health, which in turn stimulates economic development through higher productivity. In this paper, we add *CI*_*i, t*__−1_ to the model to address the endogeneity issue. These variables are then added to the estimating equation to construct the following panel threshold model of (1)–(3).

$$\begin{array}{l} Model(1):c_{it}=\mu_{it}+\beta_{1}GDP_{it}\left(GDP_{it}\leq \gamma \right)\\ \;+\beta_{2}GDP_{it}\left(GDP_{it}>\gamma \right)+\alpha_{1}^{\prime }CP_{it}\\ \;+\alpha_{2}^{\prime }IC_{it}+\alpha_{3}^{\prime }SP_{it}+\alpha_{4^{\prime }}c_{i,t}-\;1\\ Model(2):c_{it}=\mu_{it}+\beta_{1}GDP_{it}\left(GDP_{it}\leq \gamma \right)\\ \;+\beta_{2}GDP_{it}\left(GDP_{it}>\gamma \right)+\alpha_{1}^{\prime }CP_{it}\\ \;+\;\alpha_{2}^{\prime }IC_{it}+\alpha_{3}^{\prime }SP_{it}+\alpha_{4}^{\prime }c_{i,t-1}+\alpha_{5}^{\prime }GEH_{it}\\ Model(3):c_{it}=\mu_{it}+\beta_{1}GDP_{it}\left(GDP_{it}\leq \gamma \right)\\ \;+\beta_{2}GDP_{it}\left(GDP_{it}>\gamma \right)+\alpha_{1}^{\prime }CP_{it}\\ \;+\;\alpha_{2}^{\prime }IC_{it}+\alpha_{3}^{\prime }SP_{it}+\alpha_{4}^{\prime }c_{i,t-1}+\alpha_{5}^{\prime }GEH_{it}\\ \;+\;\alpha_{6}^{\prime }IP_{it} \end{array}$$

Table 6 display that for different empirical models, there is still a single threshold effect for the BRICS and ASEAN countries. According to Table 7, the estimated coefficients of economic growth for the two organizations are positive and negative in line with Table 4, indicating that there is indeed a non-linear relationship for the ASEAN countries. The robustness test shows that even with the addition of different control variables, the threshold effect of the economic growth rate is also significant. These consistent with the results in Table 3 and a more reliable conclusion is obtained.

**Table 6:** Tests for threshold effects between GDP and Health indexes.

		**Single threshold effect test**			**Double threshold effect test**			
		**Threshold value**	***F*-statistics**	***p*-Value**	**Threshold value**		***F*-statistics**	***p*-Value**
BRICS	Model (1)	0.0647^*^	9.3591	0.0715	0.0647	0.0731	8.0547	0.1532
	Model (2)	0.0636^*^	8.8915	0.0800	0.0636	0.0726	7.3599	0.1667
ASEAN	Model (1)	0.0357^**^	12.9810	0.0472	0.0357	0.0445	4.9040	0.1900
	Model (2)	0.0349^*^	11.0921	0.0589	0.0349	00491	5.2300	0.1762

** , and

* *, respectively, indicate significance at the 5, and 10% level*.

**Table 7:** Estimated coefficients of models.

		**Coefficient**	**Estimated value**	**OLS se**	***t_***OLS***_***	**White se**	***t_***White***_***
BRICS	Model (1)	${\hat{\beta }}_{1}$	−0.2891	0.1450	−1.9938*	0.1616	−1.7890*
		${\hat{\beta }}_{2}$	−0.1003	0.0730	−1.3740	0.0671	−1.4948
	Model (2)	${\hat{\beta }}_{1}$	−0.2873	0.1095	−2.6237***	0.1941	−1.4802
		${\hat{\beta }}_{2}$	−0.1003	0.0739	−1.3572	0.0632	−1.5870
	Model (3)	${\hat{\beta }}_{1}$	−0.2852	0.0731	−3.9015***	0.0929	−3.0699***
		${\hat{\beta }}_{2}$	−0.0957	0.0310	−3.0870***	0.0478	−2.0020**
ASEAN	Model (1)	${\hat{\beta }}_{1}$	−0.1290	0.0803	−1.6065*	0.0680	−1.8971*
		${\hat{\beta }}_{2}$	0.0943	0.0721	1.3079	0.0925	1.0195
	Model (2)	${\hat{\beta }}_{1}$	−0.1204	0.0599	−2.0100**	0.0517	−2.3288***
		${\hat{\beta }}_{2}$	0.1076	0.0620	1.7355*	0.0540	1.9926**
	Model (3)	${\hat{\beta }}_{1}$	−0.1292	0.0419	−3.0835***	0.0401	−3.2219***
		${\hat{\beta }}_{2}$	0.1392	0.0502	2.7729***	0.0593	2.3473***

OLS se (White se) refers to homogeneous (heterogeneous) standard deviations. ***, **, and *, respectively, indicate significance at the 1, 5, and 10% level.

The health care systems in BRICS and ASEAN countries face enormous challenges due to the demographic transition that accompanies rapid economic growth in emerging economies, which leads to an unclear relationship between business cycles and population health. However, the relevant literature does not provide convincing conclusions. Therefore, this paper uses PTRM to explore the relationship between economic growth and population health to provide more convincing conclusions. The findings suggest that the association is non-linear and asymmetric and yield a threshold economic growth rate. When economic growth is less than the estimated optimal level, there is a positive association with population health improvement. Conversely, when the level of economic development is higher than the estimated threshold, excessive health care investment leads to a waste of resources, which deteriorates population health. Therefore, it is important to keep control of the economic growth rate. Maintaining the optimal rate of economic development and actively intervening in the health care market when the economy is overheated are issues that policymakers need to address.

This study also has some limitations, so we also make some suggestions for future research. First, there is more cooperation in trade and services between BRICS and ASEAN countries, but there is also a promising cooperation and exchange in medical technology. Until this process is completed, more accurate conclusions will be drawn. Second, the key macro indicators of national health are heavily subsidized by individual countries here. However, these data are opaque, so we should pay more attention to the detailed information released by local governments. Third, as the level of medical care continues to evolve, indicators measuring the health of the population will be constantly updated, especially in the COVID-19 period. Future studies could follow this process and repeat the analysis if necessary.

## Conclusions

This paper performs a panel unit root test to examine whether Gibrat's law applies to economic growth in emerging economies. Through Table 2, we notice the stationary hypothesis of cancer incidence cannot be rejected, which suggests that health indicators follow a random walk and the rate of economic growth is the important variable effect on population health. Subsequently, this paper uses the PTRM model to explore the existence of a threshold effect of economic growth on population health in the BRICS and ASEAN countries with panel data from 1990 to 2019. In our study, economic growth is defined as real GDP per capita (GDP), and we find 0.0649 and 0.0357 as the threshold rates of economic growth in BRICS and ASEAN countries, respectively. When economic growth is below this level, more funding for health care triggers improvements in population health. However, once this level is exceeded, increased health care investment may do not receive a corresponding return. The conclusions offer worthy insights into the means of intervention by policymakers in the healthcare sector in the face of economic overheating. Changes in economic expansion may have serious negative impacts on health care systems in emerging economies, and BRICS governments should adopt more aggressive health care regulatory policies to avoid wasting health care resources during economic overheating. Population health in ASEAN countries responds more clearly during economic overheating, and policymakers can utilize their geographical advantages to actively promote intra-regional medical exchanges and mitigate the impact of the inflection point.

## Data Availability Statement

The original contributions presented in the study are included in the article/supplementary material, further inquiries can be directed to the corresponding author/s.

## Author Contributions

C-WS: conceptualization, methodology, and software. S-WH: data curation and writing- original draft preparation. RT: visualization and investigation. MH: writing-reviewing and editing. All authors contributed to the article and approved the submitted version.

## Conflict of Interest

The authors declare that the research was conducted in the absence of any commercial or financial relationships that could be construed as a potential conflict of interest.

## Footnotes

^1^BRICS countries include: Brazil, Russia, India, China and South Africa. ASEAN countries include: Indonesia, Malaysia, Philippines, Thailand, Singapore, Brunei, Cambodia, Laos, Myanmar and Vietnam.
